# Supplementary material for: Initial data analysis for longitudinal studies to build a solid foundation for reproducible analysis
Source: PLoS One. 2024 May 29;19(5):e0295726. doi: 10.1371/journal.pone.0295726 (PMC11135704; doi:10.1371/journal.pone.0295726)
Supplement: S1 File — Detailed description of the IDA plan. (PDF) [file pone.0295726.s001.pdf]

Initial data analysis plan: Age-associated decline in grip strength in the SHARE study (Denmark)

Research question: assessing the age-associated decline of hand grip strength by sex, after adjusting for a set of explanatory variables that are known to be associated with the outcome (weight, height, education level, physical activity and smoking).
Study population: individuals from Denmark aged 50 or older at baseline interview.
Variables:

Outcome variable: max grip strength

Time metric for analysis: age

Time metric for IDA: wave, measurement occasion

Time-fixed explanatory variables: sex, education, height

Time-varying explanatory variables: weight, smoking status, vigorous physical activity, moderate physical activity.

Structural variables: sex, type of interview (baseline vs. longitudinal), age groups (50 to 59, 60-69, 70-79, 80+)

Topic	Item	Features
**IDA screening domain: Participation profile**		
Time frame	P1	Calendar time, wave: (i) summarize the times when interviews were taken by calendar time, stratified by wave; (ii) evaluate the time between interviews in successive waves; (iii) describe the number of baseline and longitudinal interviews by wave; (iv) summarize the use of baseline and longitudinal questionnaires across waves and by measurement occasions.
Purpose:		(i) explore when the study was carried out and how the measurements were distributed across waves; (ii) describe time between successive measurements and the variability of the time differences; (iii) summarize the distribution of the baseline interviews vs longitudinal interviews by wave; (iv) understand the design of the study, identify possible errors in the implementation of the study (incorrect use of questionnaires).
Time metric	P2	Age: describe the distribution of age (i) stratified by wave, (ii) by type of interview (baseline or longitudinal/SHARELIFE), (iii) by sex; (iv) by wave of inclusion.
Purpose		Describe the distribution of age; explore the characteristics of the refreshment samples and the ageing of the sample during the study.

Participants	P3	Overall number of participants and number of interviews per participant.
Purpose		To evaluate the sample size and availability of repeated measurements for each participant, which can influence the complexity of the modeling of individual trajectories.
**IDA screening domain: Missing data**		
Non-enrollment	M1	Proportion of participants that were selected but did not participate in the study.
Purpose		Understand the magnitude of number of participants that were selected but did not participate.
Drop-out and intermittent missingness	M2, M3	(i) number of participants at baseline and at each successive measurement occasion, stratified by baseline wave (tables and ribbon plots to visualize the loss of participants);<br>(ii) number of participants at each measurement occasion by reason of missingness (death, out of sample, intermittent missingness, missing/definitive missing, administrative sampling, out of household);<br>(iii) comparison of baseline characteristics of participants classified by type of missingness (complete responders, deaths, intermittent missingness, lost to follow-up (out of sample or missing), out-of-household);<br>(iv) number/proportion of participants for which the vital status is unknown, and check the consistency of the information included in the data set (i.e., other possible aspects identified during data exploration, characteristics of out-of-sample and out-of-household participants).
Purpose		(i) To understand the attrition magnitude and mechanism; (ii) to summarize the number of deaths during follow-up; to differentiate types of missingness; (iii) to describe baseline characteristics of participants with different types of missingness; (iv) to evaluate the data quality.
Variable (item) missingness	M4	(i) number and proportion of missing values for the outcome and the explanatory variables (weight, smoking status, vigorous physical activity, moderate physical activity, sex, height, education) at baseline, overall and stratified by sex, wave and age group; (ii) number and proportion of missing values at all time points for the outcome and the time varying variables (weight, physical activity), overall, by type of interview, wave, measurement occasion, and by sex and age group. For the summaries stratified by measurement occasion, remove the observations where the variables are missing by design; (iii) reasons for missing outcome.

Purpose:		To evaluate the amount of missingness and obtain insights about the missing data mechanism; for the explanatory variables: to evaluate if imputation is needed, to assess from data if some variables are missing by design (for specific types of interviews/waves); for the outcome: to gain insights about the missing data mechanism. The summary of missingness by wave are useful for visualizing possible heterogeneity in data collection across waves.
Patterns	M5	Describe the co-occurrence of missing values a (i) for variables at baseline; (ii) for time-varying explanatory variables across measurement occasions, including the outcome.
Purpose		To evaluate magnitude of co-occurrence of missing values, and to evaluate patterns of co-occurrence of missingness across measurement occasions.
Non-enrollment	ME1	Compare the baseline distribution of age, sex, education of the participants that entered the study with those from the target population in EUROSTAT, stratifying by wave and type of refreshment sample.
Purpose		To evaluate if the survey participants are representative for the target population.
Probability of drop-out	ME2	Estimate the probability of loss to follow-up (LTF), death and death after loss to follow-up; stratify by sex only, and by sex and age group. Estimate cumulative incidence functions (Aalen-Johansen) for LTF and deaths, and estimate the probability of death after LTF (Kaplan-Meier). Details: define loss to follow-up (LTF) as missing data due to: out of sample, definitive missingness, out of household if not re-included in the analysis later; the estimates are obtained estimating cumulative incidence functions using Aalen-Johansen estimators for LTF and deaths (defining death times/events only for those that are not lost to follow-up - as if LTF was an absorbing state), and with Kaplan-Meier estimator to estimate the probability of death after loss to follow-up (time of entry=the time of LTF, time of end=death, time of censoring = the end of the study for those who are not dead).
Purpose		Evaluate the probability of loss to follow-up and death during the study and their association with sex and age; to understand mechanism for missing values; to check appropriateness of the methods used for handling missing values in the models.
Dropout effect on outcome	ME3	Mean profiles of outcome (grip strength) by measurement occasion, (i) stratified by drop-out measurement occasion due to death and (ii) stratified by drop-out measurement occasion due to loss to follow-up. All are stratified by sex and age group.

Purpose		To evaluate if the trajectories of the outcome variable of participants that died (were lost to follow-up) differ from those that survived (were not lost to follow-up), comparing age and sex groups; to provide empirical evidence of possible associations between the outcome and the dropout mechanism.
**IDA screening domain: Univariate descriptions**		
Description of the variables at baseline	U1	Descriptive statistics of the outcome variable and explanatory variables. For continuous variables: mean, median, interquartile range, standard deviation, high-resolution histograms, boxplots and dotplots for numerical variables. For categorical variables: number and proportion for each category graphical summaries.
Purpose		to compare the distributions with expectations and to identify possible problems in the data, e.g., digit preference, values that are present too often, , sparse categories, unexpected/unadmissible values.
Description of the time-varying variables at later points	U2	Summarize the outcome variable and the time-varying explanatory variables at later time points, using wave as time metric.
Purpose		To summarize the distribution of variables during follow-up; to identify systematic differences across waves, e.g., due to data collection issues.
**IDA screening domain: Multivariate descriptions**		
Association at baseline	V1	Visualize the association of each explanatory variable with age at baseline and sex; use age as a continuous variable and grouped in 10-year intervals
Purpose:		To check expectations about the association between explanatory variables and age and sex; to evaluate strength of the association.
Correlation at baseline	V2	Pearson correlation between all explanatory variables at baseline as heatmaps, stratified by sex.
Purpose:		To evaluate the strength of correlation between explanatory variables, which might influence the choice of the explanatory variables.
Interactions at baseline	V3	(i) explore the association between age and weight, stratified by physical activity.
Purpose		(i) The main interest will be in the interpretation of the interaction between sex and functions of age. (ii) to provide information to domain experts about possible interactions between age and status of vigorous/low intensity activity with respect to weight.
Stratification	VE1	Stratify univariate descriptions of explanatory variables by (i) sex and age group and (ii) by baseline wave

Purpose		(i) To explore the distribution of variables in subgroups of participants and (ii) to evaluate the possible differences between refresher samples
**IDA screening domain: Longitudinal aspects**		
Profiles	L1	(i) Visualize the individual profiles of the outcome for all participants, using age and measurement occasion as time metrics, stratified by sex; (ii) profile plots for subgroups of participants (10-100 per group, stratified by sex and age groups) since number or participants is too large; (iii) interactive plots that highlight individual profiles. Line colors to reflect the value of the initial grip strength.
Purpose		To describe longitudinal pattern and individual variability; these explorations can guide the choice of the functional forms of age (=time metric).
Trends	L2	Describe the longitudinal trends of the outcome variable, stratifying by sex using different time metrics (wave, measurement occasion, age). Histograms and boxplots can be used for graphical summaries, tables with univariate descriptions for numerical summaries.
Purpose		To understand average changes over time in the outcome, to evaluate the appropriateness of the functional form of time metric.
Correlation and variability	L3	Using different time metrics (wave, measurement occasion, age), estimate (i) Pearson's correlation of the outcome variable within each participants across time points, using complete pairs, and (ii) the variability of the outcome variable across time points, overall and stratified by sex.
Purpose		To get insights on (i) the correlation structure and (ii) the variance structure that will be used in modeling. Using waves we can identify some systematic errors due to wave, while measurement occasion/age is more directly related to the research question.
Trends of time-varying explanatory variables	L4	Describe numerically or graphically the longitudinal trends of the time-varying variables, using (i) wave, (ii) measurement occasion and (ii) age as time metrics, stratifying the analyses by sex. Report the overall characteristics using descriptive statistics at each time point; for physical activities variables focus on individual changes, using Sankey plots.
Purpose		To describe the average changes over time of the time-varying explanatory variables, and compare the observed trends with the expectations.
Cohort/Period effects	LE1	(i) Define birth cohort (grouped in ten year intervals, larger intervals can be used in case of sparse categories) and evaluate its association with age and wave; (ii) summarize possible cohorts on the outcome, and on the explanatory variables (iii) summarize possible cohort effect on exploratory variables.

Purpose	To assess if the variation of the outcome / exploratory variables can occur because of birth cohort effects and to guide the choice to include birth cohort as an explanatory variable in the regression model.

Table 1. Initial Data Analysis Plan for domains related to data screening in longitudinal studies for the case study
