# Supplementary material for: Initial data analysis for longitudinal studies to build a solid foundation for reproducible analysis
Source: PLoS One. 2024 May 29;19(5):e0295726. doi: 10.1371/journal.pone.0295726 (PMC11135704; doi:10.1371/journal.pone.0295726)

Additional figures and tables for the paper: Initial data analysis for longitudinal studies to build a solid foundation for reproducible analysis

by Lara Lusa, Marianne Huebner, Carsten O. Schmidt, Katherine J. Lee, Saskia le Cessie, Mark Baillie, Frank Lawrence, Cécile Proust-Lima, on behalf of TG3 of the STRATOS Initiative

Table 1. Correlation between baseline variables, overall

	Sex	Weight	Height	Education	PA Vig	PA Low	Smoking	Age
Sex	1.00	-0.48	-0.69	-0.07	-0.06	-0.02	-0.03	0.03
Weight	-0.48	1.00	0.59	0.03	0.01	0.01	-0.07	-0.18
Height	-0.69	0.59	1.00	0.18	0.13	0.06	0.01	-0.21
Education	-0.07	0.03	0.18	1.00	0.16	0.11	-0.10	-0.27
PA Vig	-0.06	0.01	0.13	0.16	1.00	0.32	-0.08	-0.26
PA Low	-0.02	0.01	0.06	0.11	0.32	1.00	-0.07	-0.22
Smoking	-0.03	-0.07	0.01	-0.10	-0.08	-0.07	1.00	-0.09
Age	0.03	-0.18	-0.21	-0.27	-0.26	-0.22	-0.09	1.00

Fig 1. Distribution of age, stratified by birth cohort and wave.

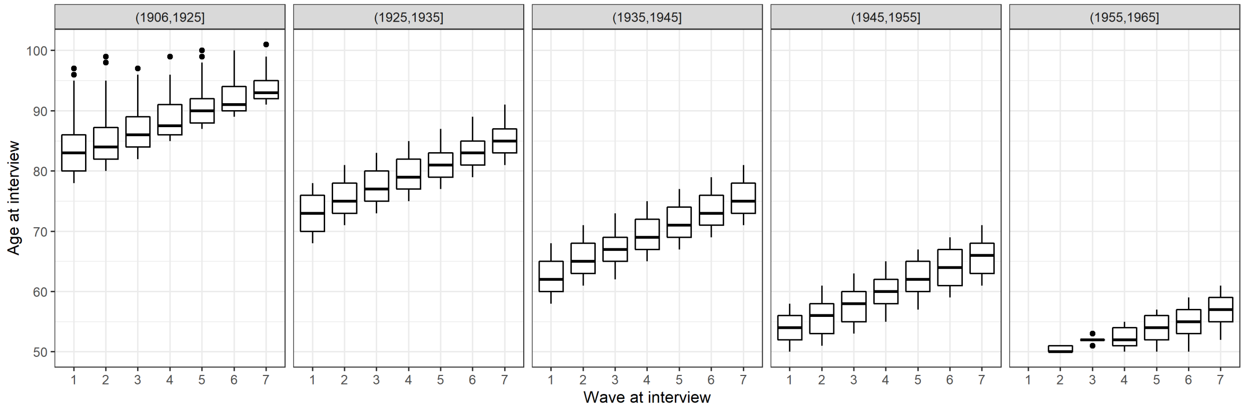

Fig 2. Profile plots of grip strength across measurement occasion, for all participants

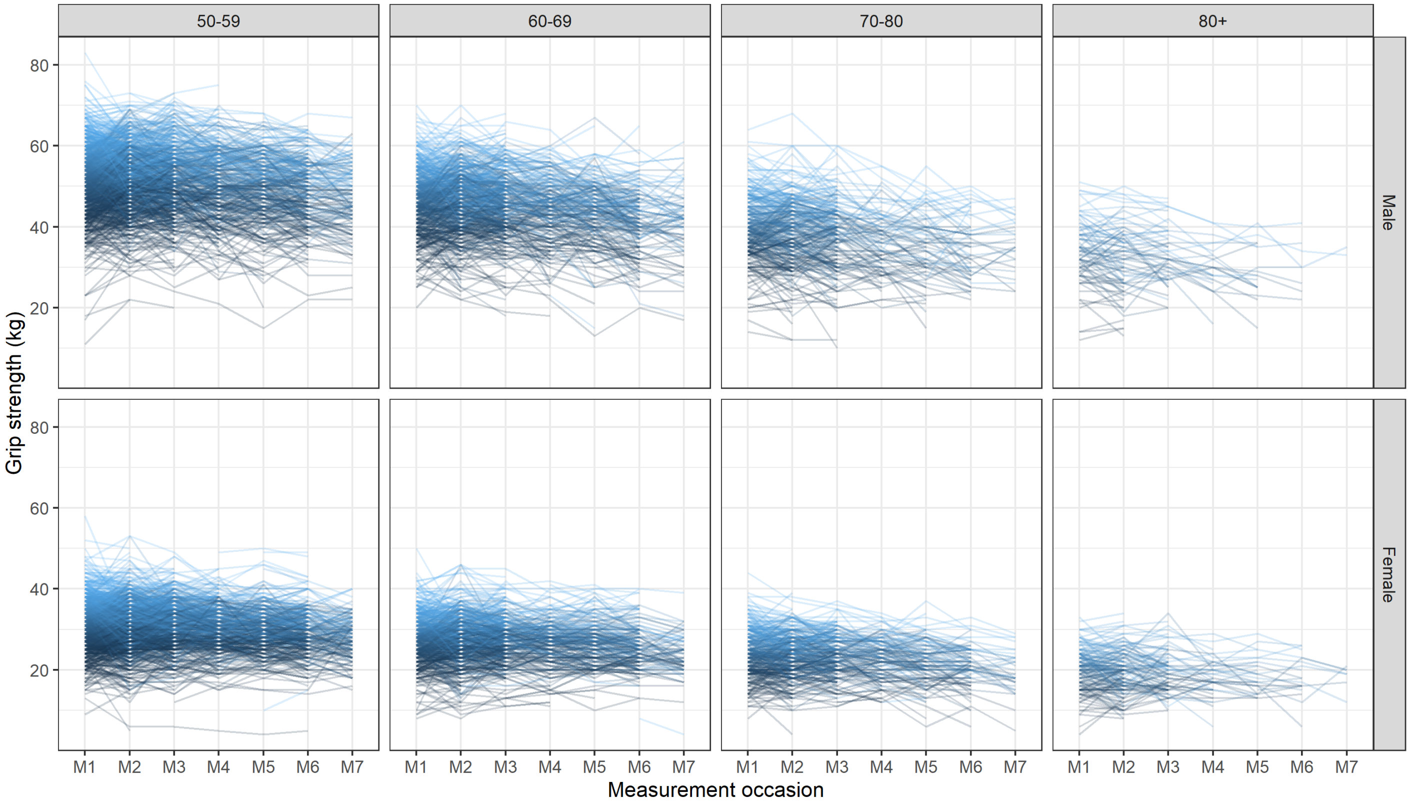

Fig 3. Profile plots of grip strength across measurement occasion, for a subset of participants (the selection of 100 participants for each group is based on the initial grip strength quantile.)

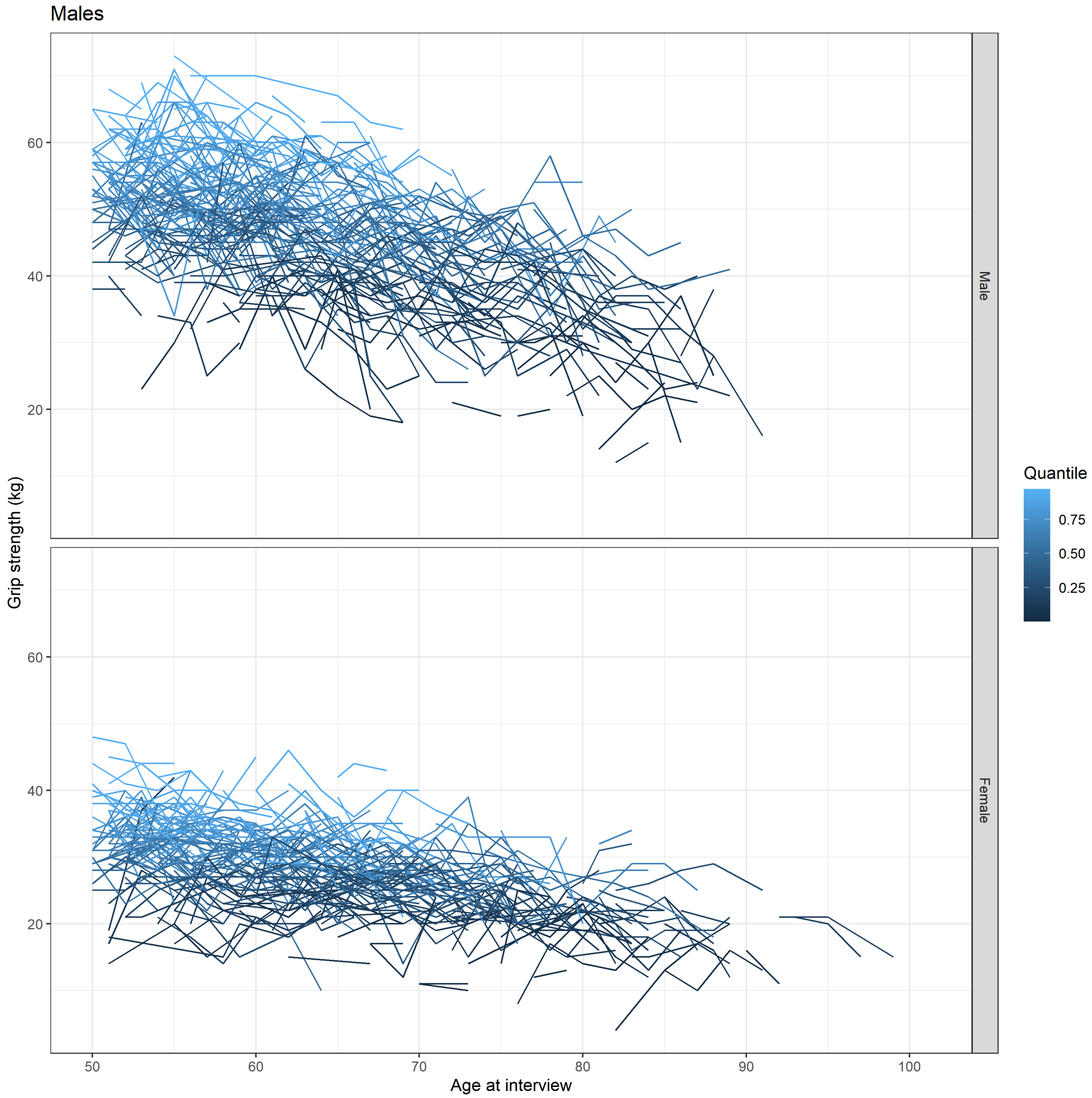

Fig 4. Scatterplot of age and weight by levels of physical activity

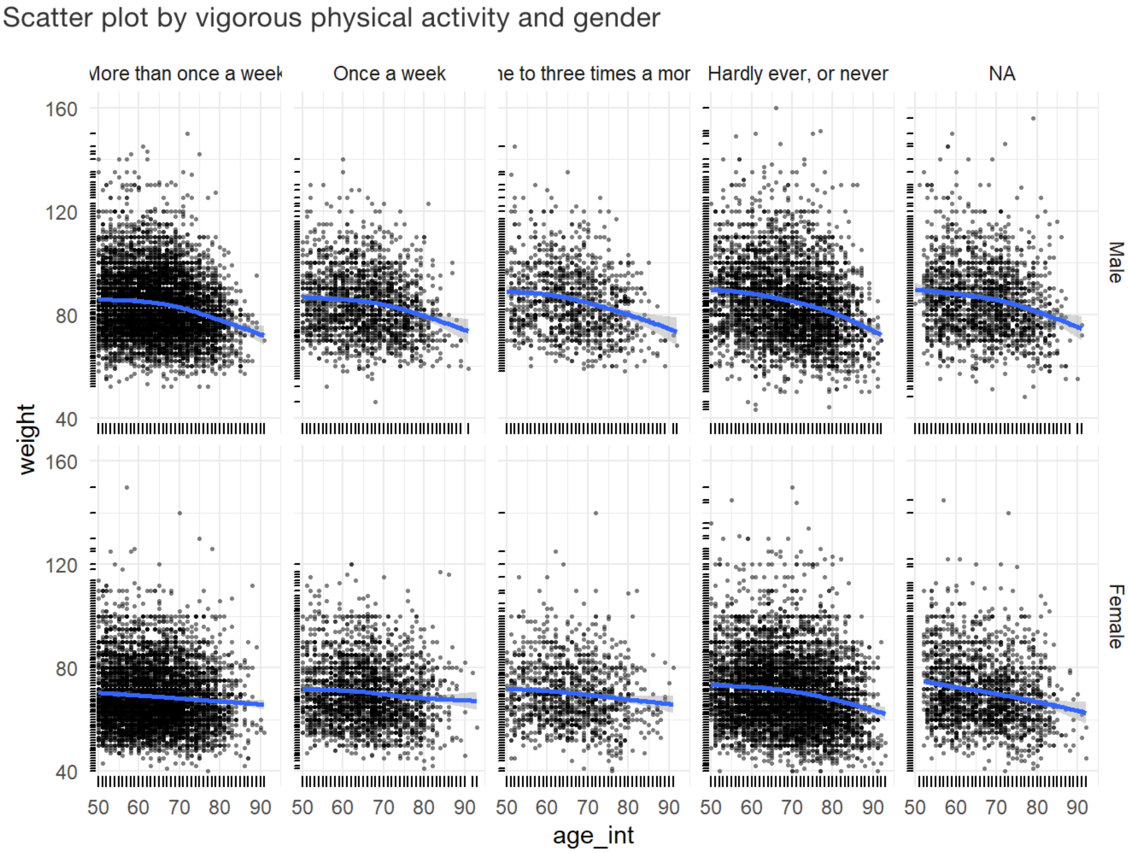

Fig 5. Average weight changes by birth cohort for males, stratified by age group. The solid black line is the estimate from the baseline data, the dashed line is estimated using all observations. The estimates are obtained using the geom_smooth() function in R.

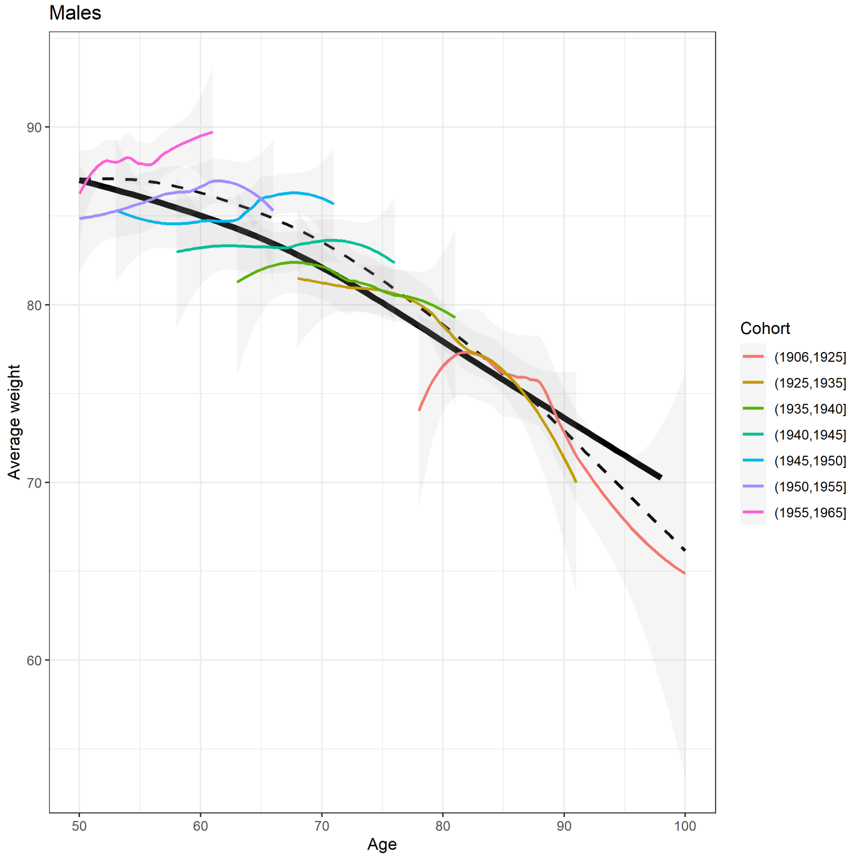

Fig 6. Proportion of participants with vigorous physical activity at least once a week by measurement occasion, by sex and age group

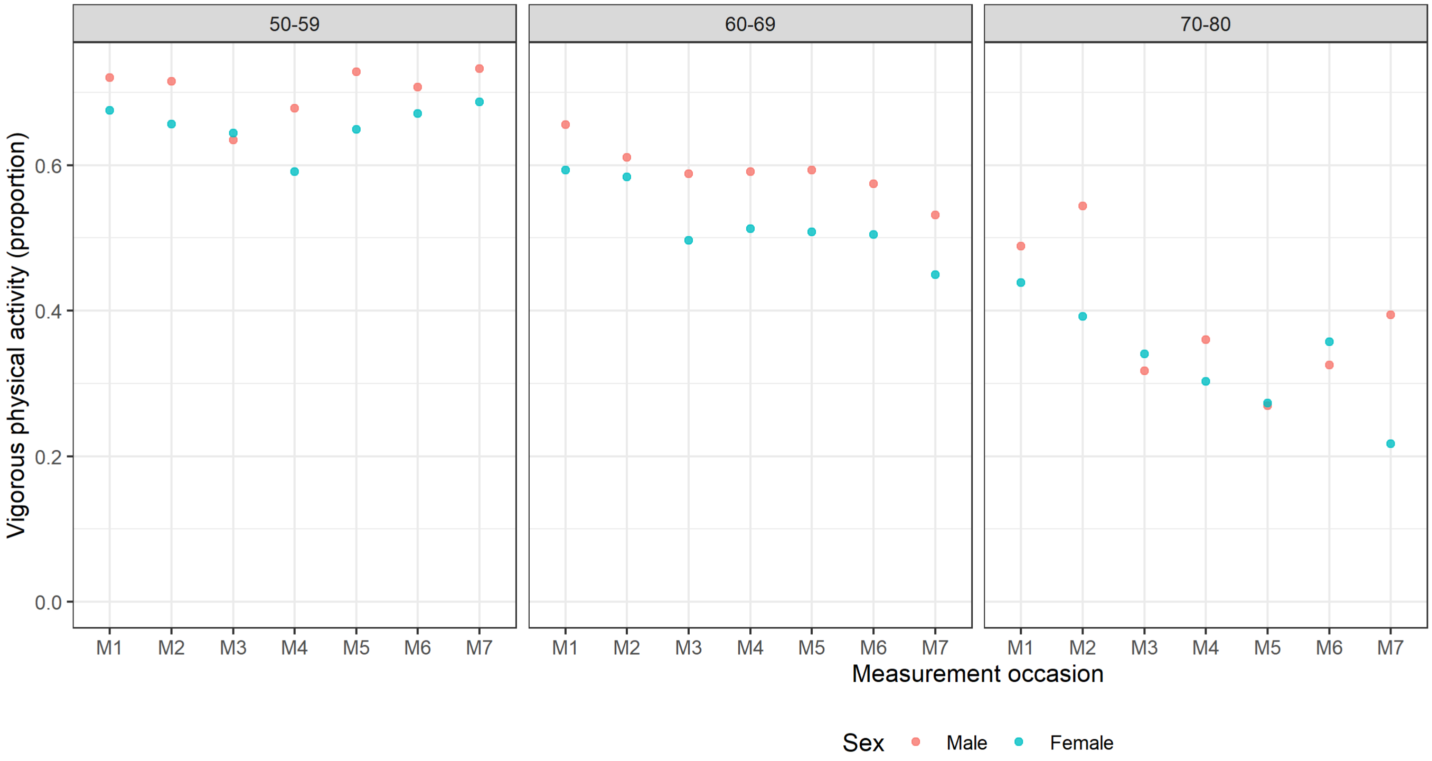

Fig 7. Parallel coordinate plots of vigorous activity status across measurement occasions (only participants with complete data in successive measurement occasions are displayed)

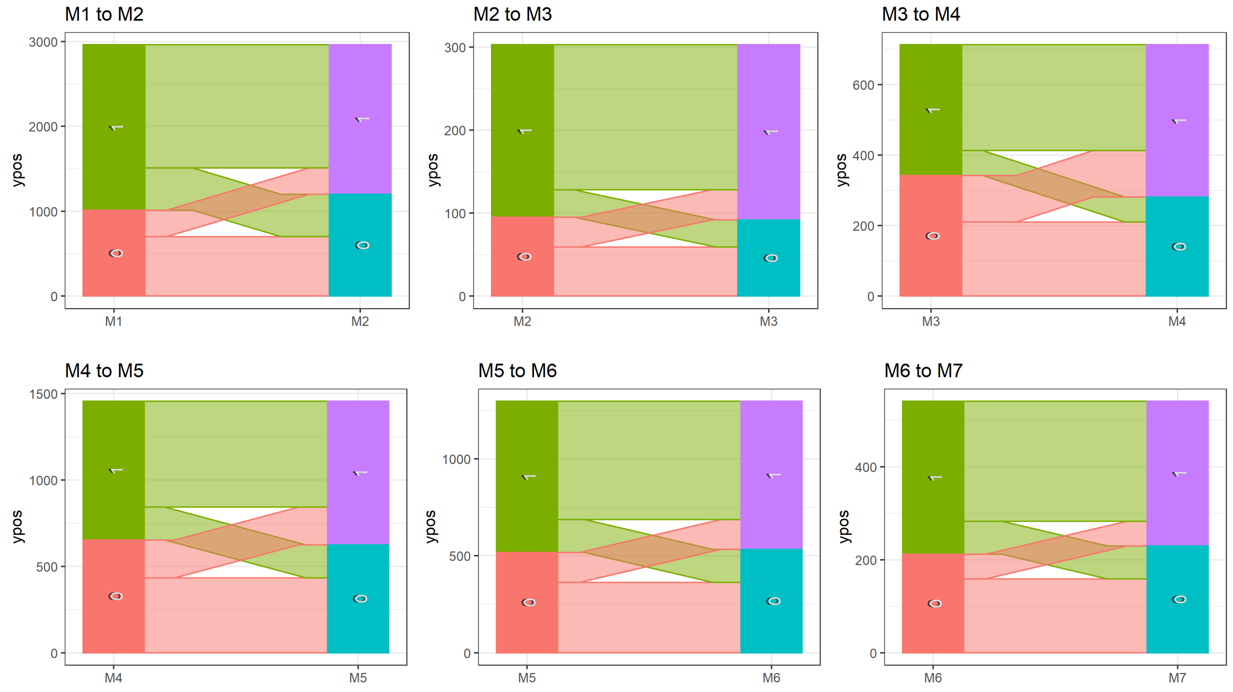

Supplement: S2 File — (PDF) [file pone.0295726.s002.pdf]
